# Identification of key biomarkers in RF-negative polyarticular and oligoarticular juvenile idiopathic arthritis by bioinformatic analysis

**DOI:** 10.1186/s12969-023-00926-4

**Published:** 2023-11-24

**Authors:** Yun Liu, Xuemei Tang

**Affiliations:** 1 Chongqing Key Laboratory of Child Infection and Immunity, Children’s Hospital of Chongqing Medical University, Chongqing, 400010 China; 2 Department of Rheumatology and Immunology, Children’s Hospital of Chongqing Medical University, Chongqing, 400010 China

**Keywords:** Bioinformatics, Biomarker, Immune infiltration, Oligoarticular juvenile idiopathic arthritis, RF-negative polyarticular juvenile idiopathic arthritis

## Abstract

**Objective:**

Juvenile idiopathic arthritis (JIA) is a broad term used to describe arthritis of unknown origin. JIA commonly persists into adulthood, often causing substantial morbidity such as restricted joint function, which can lead to challenges in employment and independence. This study aims to identify diagnostic biomarkers and investigate the role of immune cells in the pathogenesis of rheumatoid factor-negative polyarticular juvenile idiopathic arthritis (RF-negative pJIA) and oligoarticular juvenile idiopathic arthritis (oJIA).

**Methods:**

We retrieved a JIA dataset from the GEO database and conducted an analysis of differentially expressed genes (DEGs). Subsequently, functional enrichment analysis was performed on the DEGs. Weighted gene co-expression network analysis (WGCNA) was utilized to identify key modules. Additionally, we constructed a protein‒protein interaction network to identify hub genes that serve as signature genes. Furthermore, we employed CIBERSORT to classify immune cell infiltration.

**Results:**

From the GSE20307 dataset, we identified a total of 1438 DEGs in RF-negative pJIA and 688 DEGs in oJIA. WGCNA clustered the data into 6 modules in pJIA and 4 modules in oJIA. Notably, the ME5 and ME2 modules exhibited significant associations with pJIA and oJIA, respectively. In both pJIA and oJIA, we identified six hub genes, four of which demonstrated high diagnostic sensitivity and specificity in pJIA, while five showed high diagnostic sensitivity and specificity in oJIA. CIBERSORT analysis suggested the potential involvement of these signature genes in immune cell infiltration.

**Conclusion:**

In this study, we identified JUN, CXCL8, SOCS3, and KRAS as biomarkers for RF-negative pJIA and JUN, CXCL8, SOCS3, PTGS2, and NFKBIA as biomarkers for oJIA. Furthermore, our findings suggest that Tfh cells may play a role in the early onset of both RF-negative pJIA and oJIA.

**Supplementary Information:**

The online version contains supplementary material available at 10.1186/s12969-023-00926-4.

## Introduction

Juvenile idiopathic arthritis encompasses various forms of arthritis with unknown causes that manifest before the age of 16 and persist for more than 6 weeks [1, 2]. In the late 1990s, the International League Against Rheumatism (ILAR) introduced a classification system for JIA that distinguishes seven categories based on the signs and symptoms occurring within the initial six months of the disease. These categories include systemic arthritis, rheumatoid factor-positive polyarthritis, rheumatoid factor-negative polyarthritis, oligoarthritis, enthesitis-related arthritis, psoriatic arthritis, and undifferentiated arthritis [3, 4]. RF-negative pJIA occurs in 15–20% of all JIA cases, while oJIA represents 50% of JIA cases in Western populations. The PRINTO New Classification Criteria categorize RF-negative pJIA and oJIA together [5]. RF-negative pJIA and oJIA, which are specific to children, lead to joint destruction, resulting in restricted joint function and continuing into adulthood [1, 2] Additionally, some patients face a high risk of complications, such as chronic iridocyclitis, and exhibit a poor long-term prognosis [6]. Children with iridocyclitis face a potential risk of severe complications, such as posterior syndrome, cataracts, band keratopathy, macular edema, and glaucoma, ultimately resulting in visual impairment or blindness. Early diagnosis and treatment are crucial as they significantly impact outcomes. Therefore, prioritizing early detection and intervention is vital.

In recent years, progress in bioinformatics and sequencing technologies has facilitated the identification of differentially expressed genes (DEGs) linked to JIA and the investigation of its underlying mechanisms. However, there is currently limited information regarding RF-negative pJIA and oJIA. Therefore, the objective of this study was to investigate the diagnostic markers and potential therapeutic targets specific to RF-negative pJIA and oJIA. To achieve this, we utilized various tools, including the R package and Cytoscape.

## Materials and methods

### Data sources

For this study, two datasets were obtained from the Gene Expression Omnibus (GEO) (http://www.ncbi.nlm.nih.gov/geo/), specifically GSE20307 and GSE13501. Both datasets utilized the GPL570 [HG-U133_Plus_2] Affymetrix Human Genome U133 Plus 2.0 Array platform. GSE20307 was designated as the training set and comprised 140 subjects, including 56 healthy individuals, 44 RF-negative pJIA patients, and 40 oJIA patients. On the other hand, GSE13501 served as the validation set and consisted of 12 healthy individuals, 46 RF-negative pJIA patients, and 43 oJIA patients. Written informed consent was obtained from all participants, and it is important to note that all cases were newly diagnosed and had not undergone any prior drug treatment.

### Identification of Differentially Expressed Genes (DEGs)

The limma package [7] in the R software was utilized to analyze the DEGs between the RF-negative pJIA and oJIA cohorts and the control cohort. The criteria set for the analysis were a *p*_value < 0.05 and |fold change (FC)|> 1.2. A volcano plot was generated to visualize the DEGs.

### Functional and pathway enrichment analyses

Functional enrichment analyses of the DEGs based on Gene Ontology (GO) and the Kyoto Encyclopedia of Genes and Genomes (KEGG)8 were conducted using the cluster Profiler package in R [8]. The GO analysis encompassed three categories: biological process (BP), cellular component (CC), and molecular function (MF). Additionally, KEGG analysis was employed to identify potential signaling pathways.

### Weighted gene co-expression network analysis

The construction of the co-expression network in the GSE20307 cohort involved employing the Weighted Gene Co-Expression Network Analysis (WGCNA) technique based on the scale-free topology criterion [9]. The Pick Soft Threshold function of the WGCNA package determined the soft threshold power and adjacencies. The resulting adjacency matrix was transformed into a topological overlap matrix (TOM), and hierarchical clustering analysis was performed using a corresponding dissimilarity measure. A dynamic tree cutting approach with a minimum module size of 30 was employed to identify co-expression gene modules. The connection between the gene modules and children diagnosed with pJIA and oJIA was evaluated using gene significance (GS) and module membership (MM) values, which were used to identify key gene modules.

### Identification of signature genes

To construct a protein‒protein interaction (PPI) network for the key modules, the Search Tool for the Retrieval of Interacting Genes (STRING) online tool (version 11.0; http://string-db.org) was utilized. The resulting PPI network was analyzed using Cytoscape software, and the critical subnetwork and hub genes were identified using the cytoHubba plug-in.

### Immune cell infiltration

CIBERSORT, a method based on linear support vector regression, was employed to deconvolute the expression matrix of 22 human immune cell subtypes. This analysis aimed to explore differences in immune cell infiltration between children with RF-negative pJIA oJIA and healthy individuals [10]. Immune cells showing significant infiltration differences were subsequently analyzed for correlation with the signature genes using the Spearman method.

### Statistical analysis

All statistical analyses for this study were performed using R software (version 4.1.3). ROC curve analysis was conducted using the pROC R package. All statistical tests were two-tailed, and a value < 0.05 was considered statistically significant.

## Results

### Identification of Differentially Expressed Genes (DEGs) between RF-Negative pJIA, oJIA, and control

**Fig. 1:**
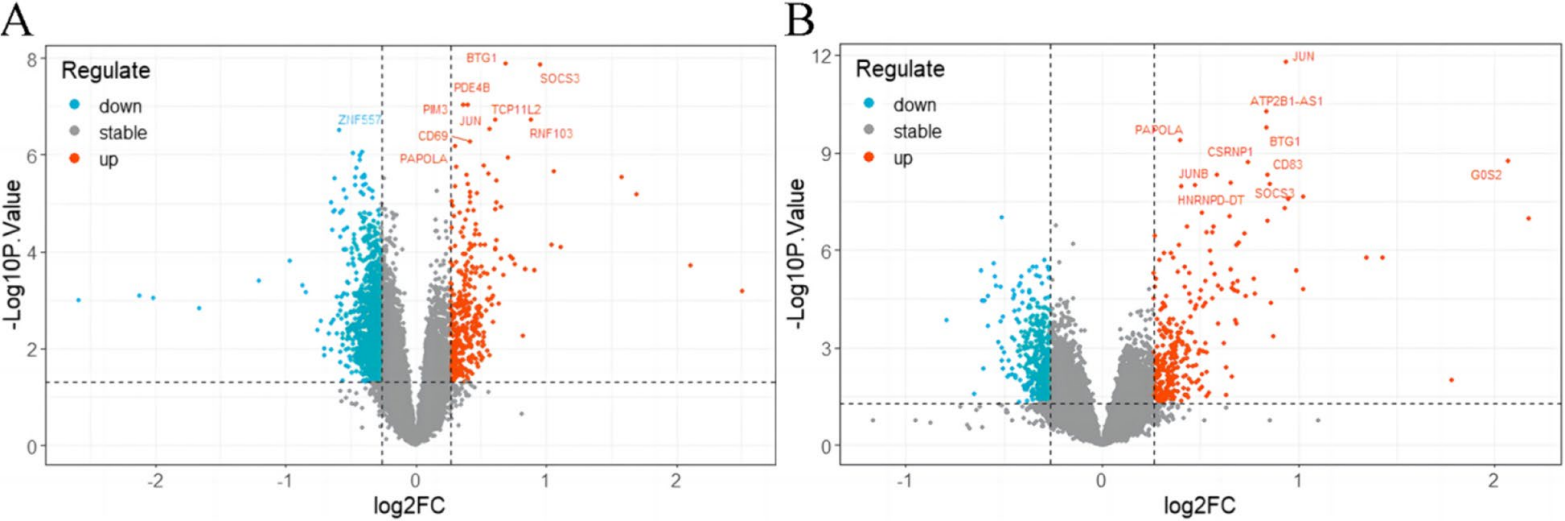
Identification of the DEGs in RF-negative pJIA, oJIA, and healthy individuals. **A** Volcano showed expression of DEGs between RF-negative pJIA and healthy individuals. **B** Volcano showed expression of DEGs between oJIA and healthy individuals

We analyzed the DEGs between children with RF-negative pJIA, oJIA, and healthy individuals using the “limma” package. A total of 1438 DEGs were identified, with 355 upregulated and 1083 downregulated genes in pJIA (Fig. 1A). Similarly, in oJIA, we found 688 DEGs, including 282 upregulated and 406 downregulated genes (Fig. 1B).

### Functional enrichment analysis

**Fig. 2:**
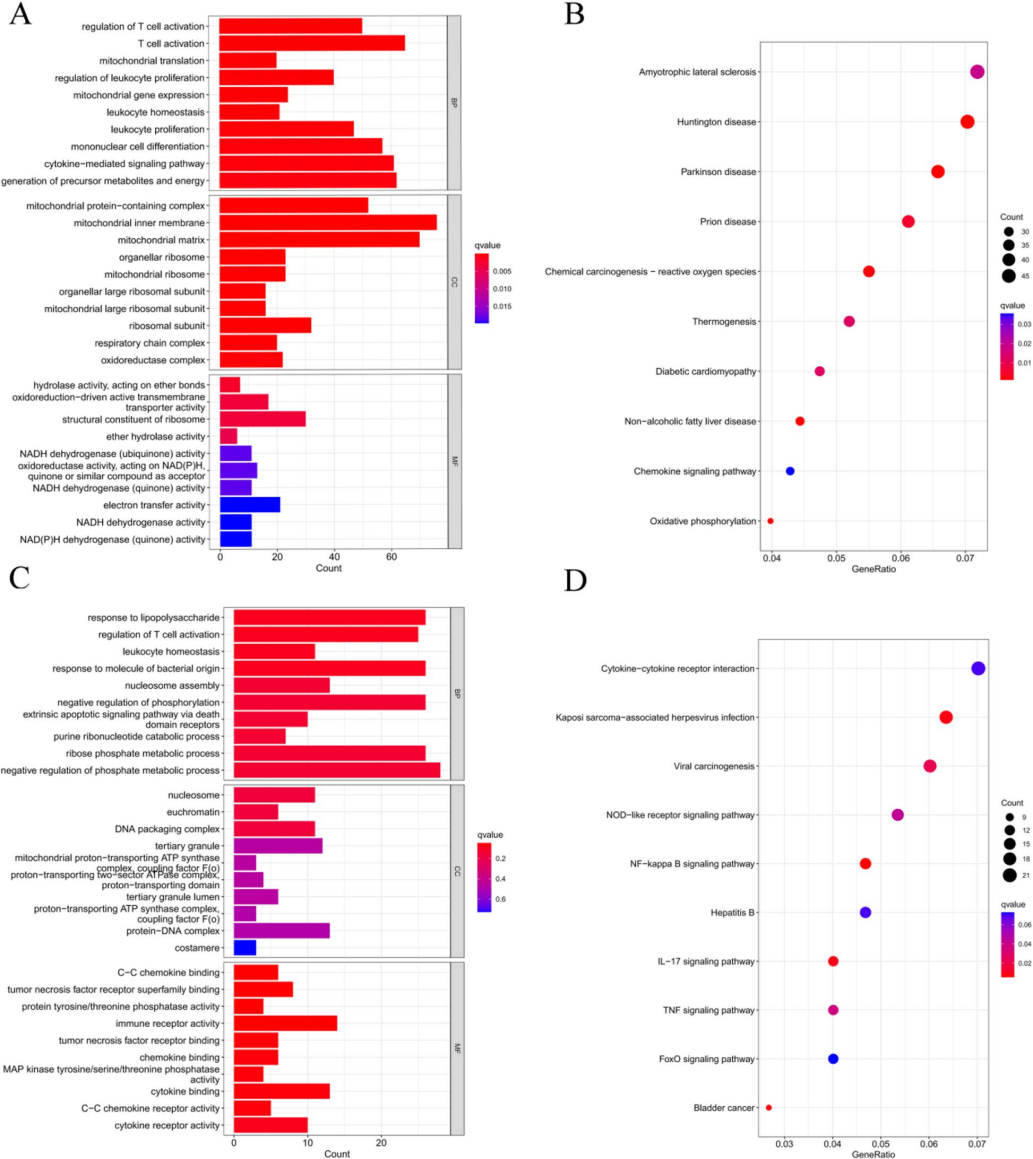
Functional enrichment analysis of DEGs. **A** The top 10 functional enrichment in BP, CC, and MF analysis between RF-negative pJIA and healthy individuals. **B** The KEGG analysis of DEGs between RF-negative pJIA and healthy individuals. **C** The top 10 functional enrichment in BP, CC, and MF analysis between oJIA and healthy individuals. **D** The KEGG analysis of DEGs between oJIA and healthy individuals

Functional enrichment analysis based on Gene Ontology (GO) and Kyoto Encyclopedia of Genes and Genomes (KEGG) was performed to understand the cellular mechanisms underlying RF-negative polyarticular juvenile idiopathic arthritis (pJIA) and oligoarticular juvenile idiopathic arthritis (oJIA). RF-negative pJIA was associated with key functions such as T-cell activation, leukocyte proliferation, mononuclear cell differentiation, and the chemokine signaling pathway (Fig. 2A, B). In oJIA, functions related to T-cell activation, leukocyte homeostasis, and the NF-kappa B, IL-17, and TNF signaling pathways were regulated (Fig. 2A, B). These functions play a role in adaptive immunity.

### Construction of the weighted gene co-expression network

**Fig. 3:**
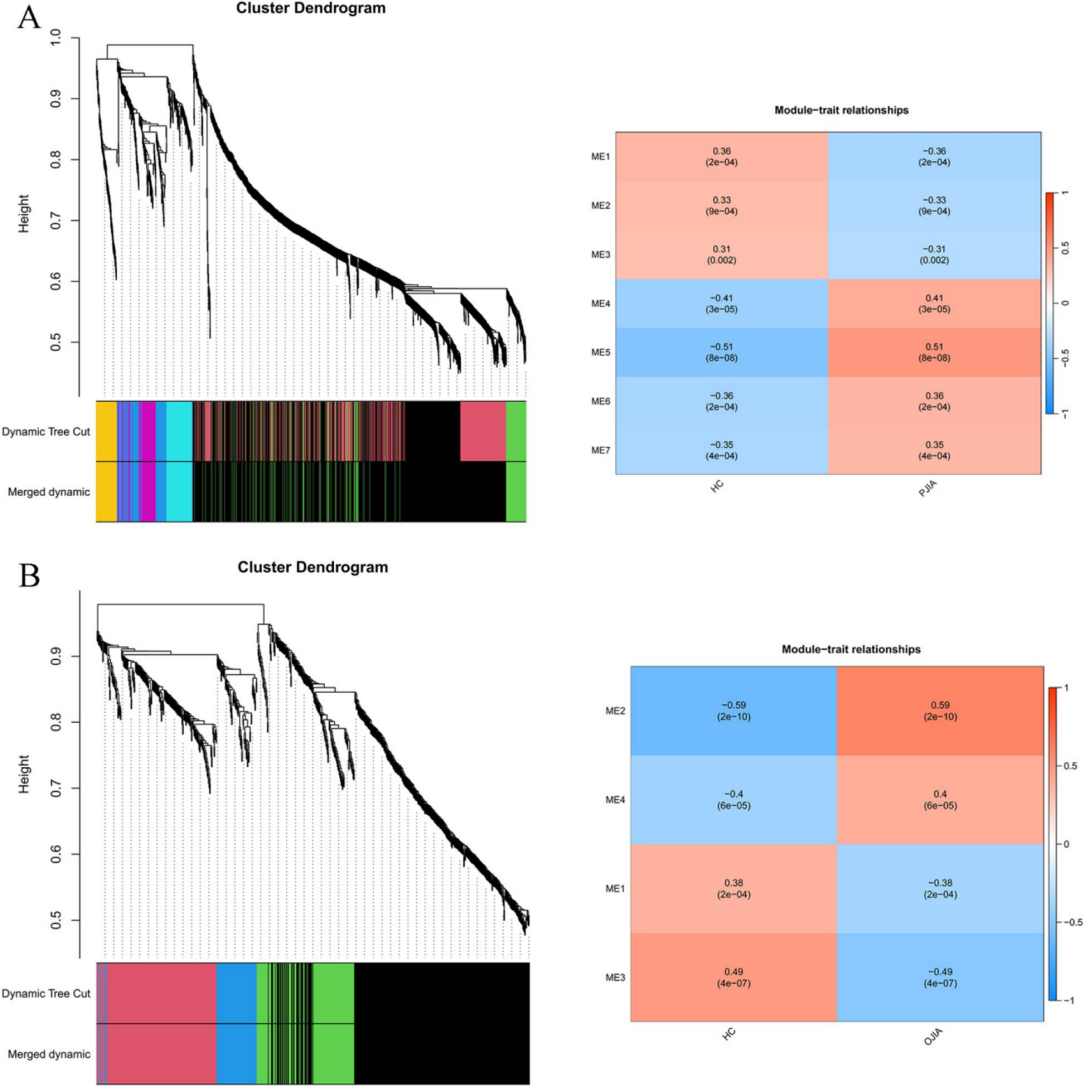
The WGCNA analysis of GSE20307. **A** The mean connectivity and the cluster dendrogram of WGCNA between RF-negative pJIA and healthy individuals. **B** The mean connectivity and the cluster dendrogram of WGCNA between oJIA and healthy individuals

**Fig. 4:**
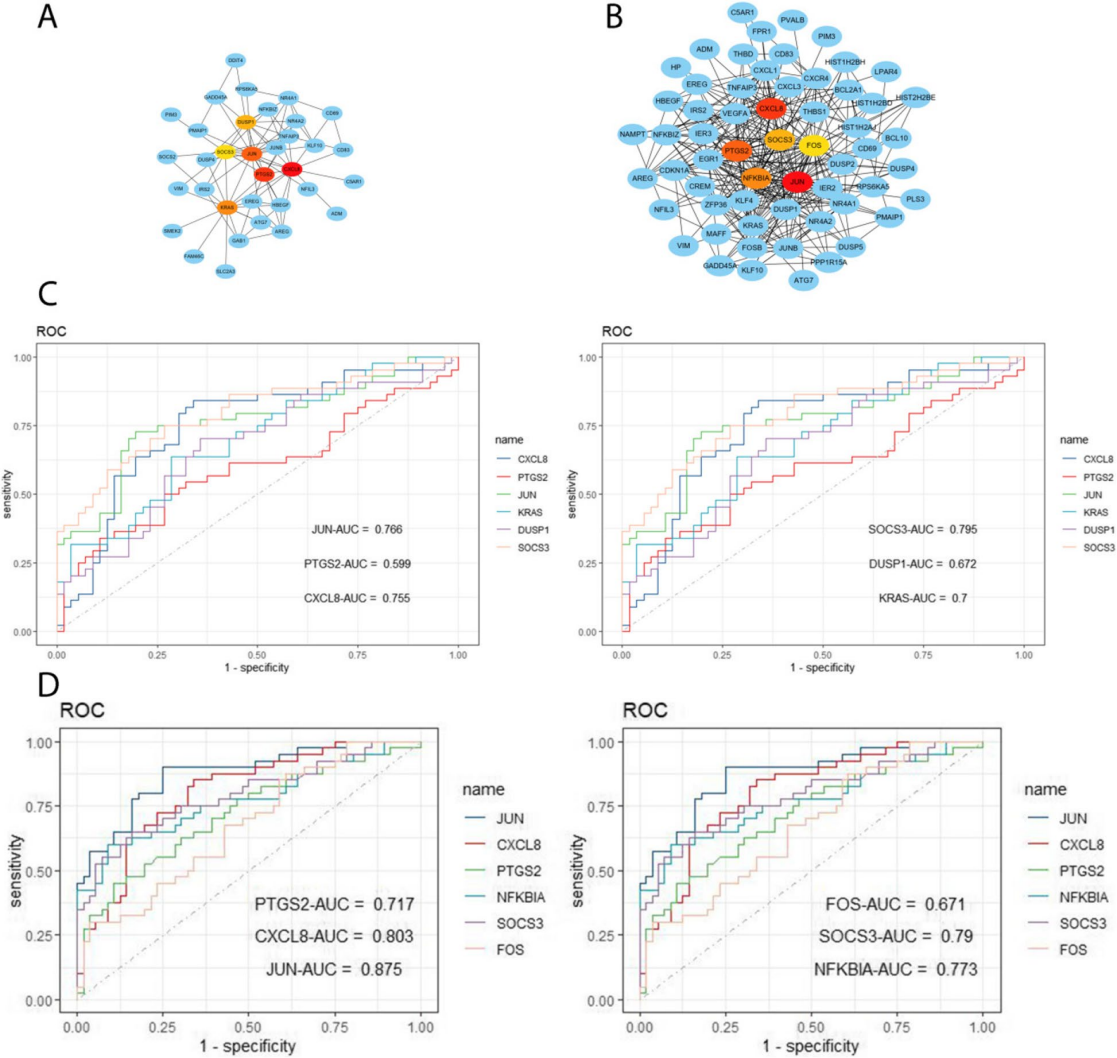
Hub gene identification and diagnosis prediction analysis. **A** Networks of the six hub genes identified by “cytoHubba” between RF-negative pJIA and healthy individuals. The yellow-to-red color scale denotes the *p*-value calculated by the MCC method. **B** Networks of the six hub genes identified by “cytoHubba” between oJIA and healthy individuals. The yellow-to-red color scale denotes the *p*-value calculated by the MCC method. **C** Diagnostic utility of the six hub genes in the GSE20307 datasets between RF-negative PJIA and healthy individuals. **D** Diagnostic utility of the six hub genes in the GSE20307 datasets between oJIA and healthy individuals

Using the WGCNA package in R software, we conducted a comprehensive analysis of RF-negative pJIA, oJIA, and healthy individuals. The initial co-expression network was established with a soft threshold power of 6, meeting the scale-free index of 0.8 and a favorable mean connectivity. The resulting cluster dendrogram (Fig. 3) revealed the formation of 6 modules in RF-negative pJIA. Among them, the ME5 module showed a significant correlation (cor = 0.51, *p* < 0.001) with RF-negative pJIA, indicating its importance in the disease’s pathogenesis. The PPI network constructed using Cytoscape software identified six hub genes, namely CXCL8, PTGS2, JUN, KRAS, DUSP1, and SOCS3, out of 87 analyzed genes (Fig. 4A).

Similarly, in oJIA, we identified 4 modules and calculated their correlations with the disease. The ME2 module exhibited a strong correlation (cor = 0.59, *p* < 0.001) with oJIA, indicating its significance. The corresponding PPI network identified six hub genes JUN, CXCL8, PTGS2, NFKBIA, SOCS3, and FOS, out of 185 analyzed genes (Fig. 4B). These findings shed light on the molecular mechanisms underlying RF-negative pJIA and oJIA, providing potential therapeutic targets.

### Diagnostic efficacy of signature genes in RF-negative pJIA and oJIA

To evaluate the diagnostic value of the ME5 module for RF-negative pJIA, we performed ROC analysis on gene candidates using the GSE20307 dataset. JUN, CXCL8, KRAS, and SOCS3 showed superior performance in distinguishing RF-negative pJIA patients from healthy individuals, with AUC values of 0.755, 0.766, 0.7, and 0.795, respectively (Fig. 4C).

Additionally, we evaluated the diagnostic potential of the ME2 module in oJIA through ROC analysis of gene candidates’ sensitivity and specificity. Our analysis of the GSE20307 dataset identified CXCL8, JUN, PTGS2, NFKBIA, and SOCS3 as having the highest diagnostic value for differentiating oJIA patients from healthy controls. Notably, these hub genes in the GSE20307 training set demonstrated good sensitivity and specificity, with AUC values of 0.803, 0.875, 0.717, 0.773, and 0.79 for CXCL8, JUN, PTGS2, NFKBIA, and SOCS3, respectively (Fig. 4D).

### Identification of overlapping hub genes and correlation with immune infiltration

**Fig. 5:**
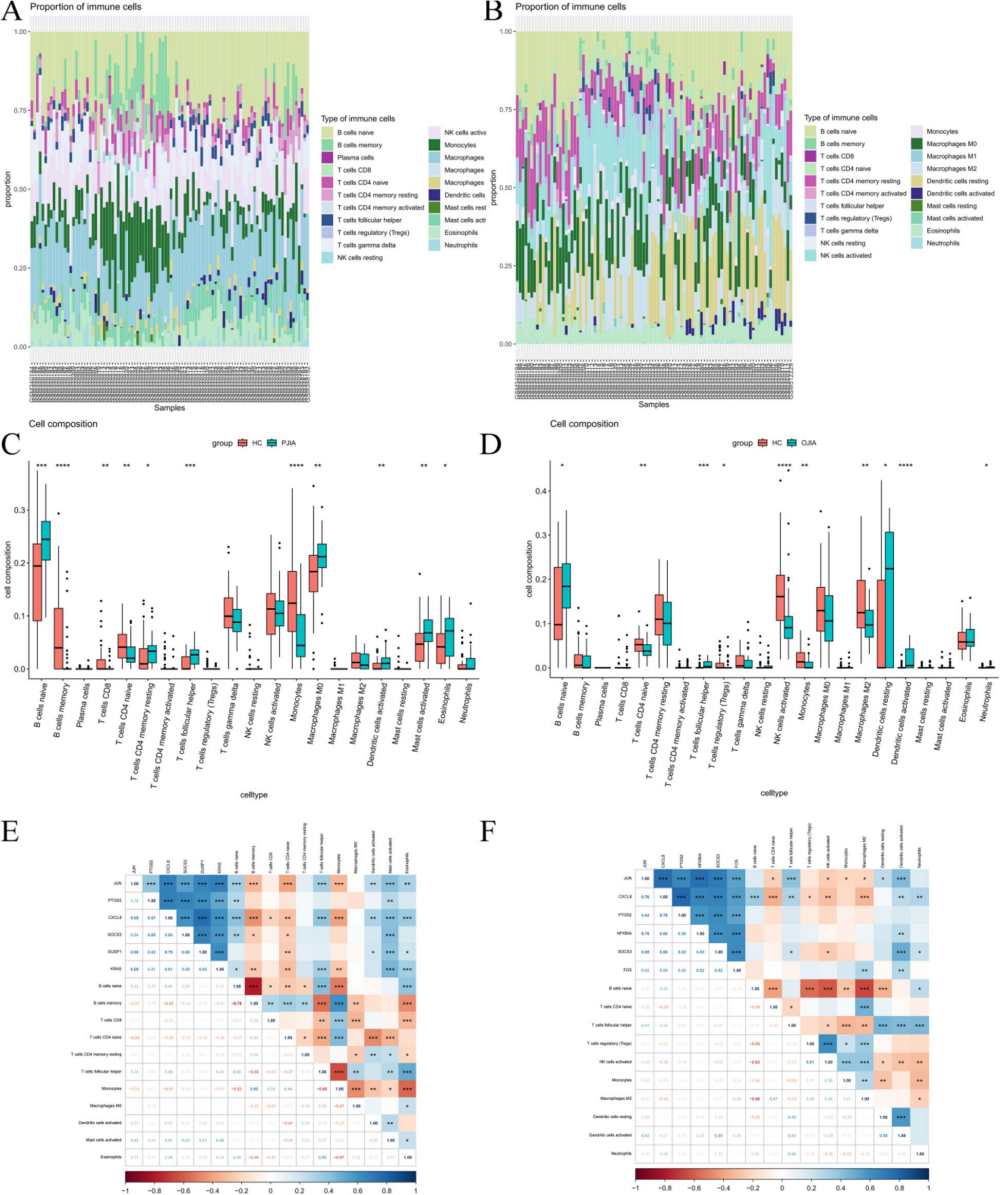
Immune cell infiltration analysis of the GSE20307 datasets. The composition of immune cells in each sample was shown in a histogram between RF-negative pJIA and healthy individuals (**A**) and between oJIA and healthy individuals (**B**). The GSE20307 datasets were analyzed to determine differences in immune cell infiltration between RF-negative pJIA and healthy individuals (**C**) and between oJIA and healthy individuals (**D**). Correlation heat maps of immune cells between RF-negative pJIA and healthy individuals (**E**) and between oJIA and healthy (**F**)

To further confirm the association between hub gene expression and immune infiltration, we utilized the CIBERSORT algorithm to assess the proportions of 22 immune cells in RF-negative pJIA (Fig. 5A) and oJIA (Fig. 5B). Our findings revealed that RF-negative pJIA patients had a higher abundance of naive B cells, resting memory CD4 T cells, follicular helper T cells, M0 macrophages, M1 macrophages, activated dendritic cells, activated mast cells, and eosinophils than healthy controls. Conversely, memory B cells, CD8 T cells, naive CD4 T cells, and monocytes were comparatively lower in RF-negative pJIA (Fig. 5C). Additionally, we observed significant correlations between five immune cell types and CXCL8 expression levels (*P* < 0.05). Specifically, naive B cells, follicular helper T cells, and mast cells exhibited positive correlations with CXCL8 expression, while memory B cells and monocytes displayed negative correlations (Fig. 5E).

Similarly, in oJIA patients, the proportions of naive B cells, follicular helper T cells, and activated dendritic cells were elevated compared to those in healthy controls, while the proportions of naive CD4 T cells, Tregs, activated NK cells, monocytes, and M2 macrophages were comparatively lower (Fig. 5D). Moreover, follicular helper T cells and activated dendritic cells showed positive correlations with JUN expression, and activated dendritic cells alone were positively correlated with SOCS3 expression (Fig. 5F). These observations suggest that CXCL8 might play a role in the pathogenesis of RF-negative pJIA, while JUN and SOCS3 may regulate immune cell infiltration in oJIA.

### Validation of hub gene expression

**Fig. 6:**
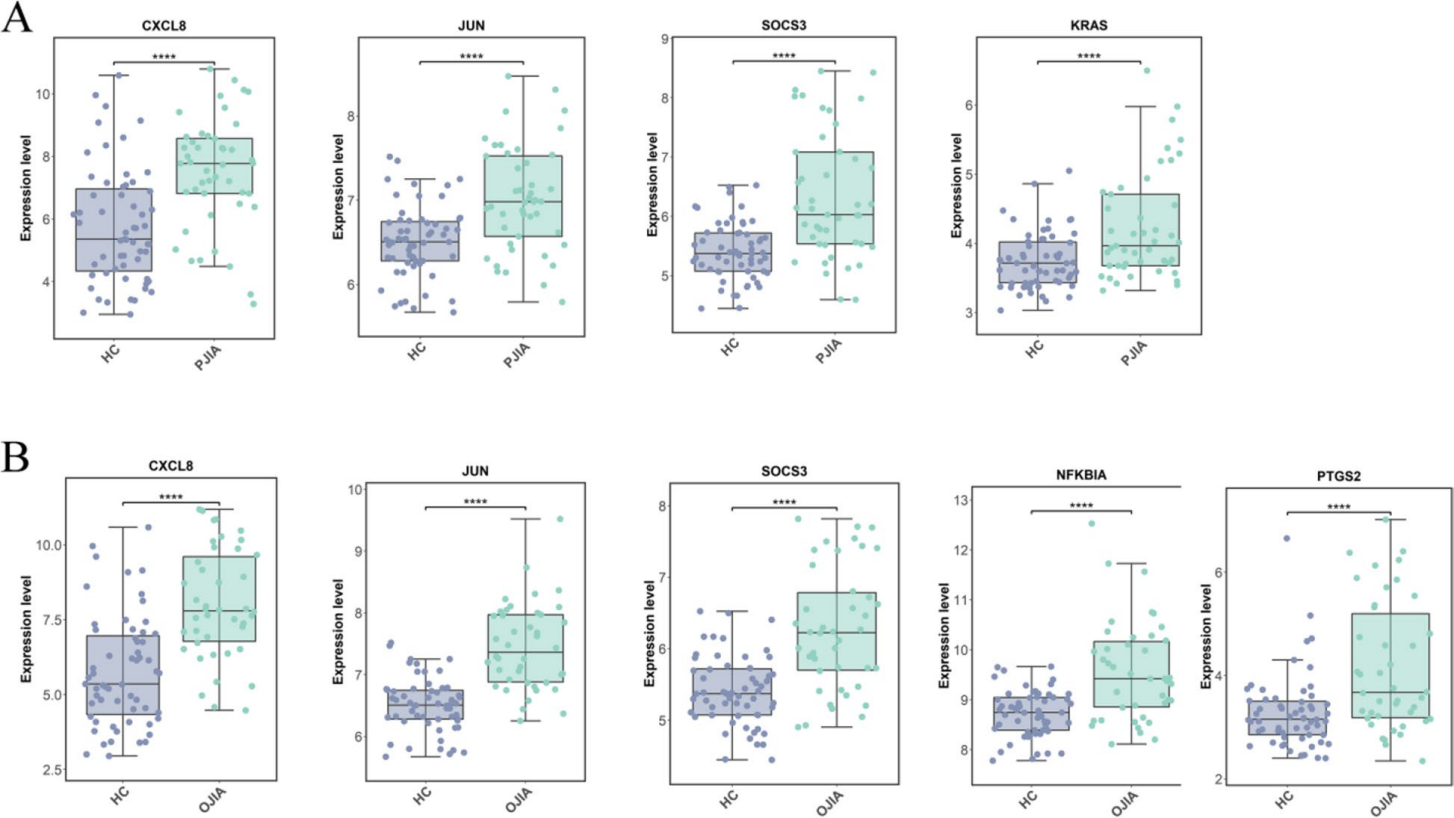
Validation of hub genes expression. **A** CXCL8, JUN, SOCS3, and KRAS expression between RF-negative pJIA and healthy individuals in dataset GSE13501. **B** CXCL8, JUN, SOCS3, NFKBIA and PTGS2 expression between oJIA and healthy individuals in dataset GSE13501

To validate the accuracy of the transcriptomic data, we conducted a validation study on hub genes using the GSE13501 dataset. The hub genes for RF-negative pJIA (JUN, CXCL8, KRAS, and SOCS3) exhibited significant differences in the validation dataset GSE13501 (Fig. 6A). Similarly, the hub genes for oJIA (CXCL8, JUN, PTGS2, NFKBIA, and SOCS3) also showed significant differences in the validation dataset GSE13501 (Fig. 6B). These findings suggest that the identified hub genes are reliable and may serve as potential biomarkers for the respective subtypes of JIA.

## Discussion

JIA is a prevalent form of arthritis among children that leads to restricted joint mobility caused by both RF-negative pJIA and oJIA. In pursuit of enabling early diagnosis, we conducted a bioinformatic analysis of two datasets, namely, GSE20307 and GSE13501, utilizing various tools. Among these, six pivotal genes were identified, out of which CXCL8, JUN, KRAS, and SOCS3 displayed high diagnostic efficacy in RF-negative pJIA, whereas JUN, CXCL8, PTGS2, NFKBIA, and SOCS3 exhibited similar performance in oJIA, as determined by ROC curve analysis. To validate our findings, we analyzed the expression levels of the hub genes in RF-negative pJIA, oJIA, and healthy controls using the GSE13501 dataset. The outcomes revealed significantly upregulated expression of CXCL8, JUN, KRAS, and SOCS3 in RF-negative pJIA patients and JUN, CXCL8, PTGS2, NFKBIA, and SOCS3 in oJIA patients compared with the control group, which is consistent with our bioinformatics analysis.

As per the PRINTO New Classification Criteria for Juvenile Idiopathic Arthritis, JIA can be classified into six distinct categories: A. Systemic JIA, B. RF-positive JIA, C. Enthesitis/spondylitis-related JIA, D. Early-onset ANA-positive JIA, E. Other JIA, and F. Unclassified JIA5. The revised classification criteria reshuffle the majority of patients who were previously categorized as oligoarthritis and included in the RF-polyarthritis and PsA categories and place them under Category D. Early-onset ANA-positive JIA, which aligns with our study findings. Our observations indicate that hub genes, including JUN, SOCS3, and CXCL8, were shared between RF-negative pJIA and oJIA. The identification of the JUN protein can be traced back to cells carrying avian sarcoma virus [11, 12] and it belongs to the proto-oncogene family [13, 14]. The c-Jun amino-terminal kinases (JNKs), which are proline-directed kinases and are activated by UV radiation and oncoproteins, have been identified [15, 16]. Similar to ERKs, JNKs preferentially phosphorylate serine and threonine located within Pro-Xaa-Ser/Thr-Pro sequences, and they participate in the MAPK pathway, which may be related to RF-negative pJIA and oJIA. SOCS3, a member of the SOCS protein family, acts as a classical negative regulator of JAK/STAT pathway signaling and consists of eight similar proteins [17, 18]. The feedback inhibition of the JAK/STAT3 pathway by the SOCS3 protein involves its binding to its corresponding receptor, the primary receptor shared interleukin-6 (IL-6) receptor subunit gp130, to inhibit STAT3 phosphorylation [19–21]. SOCS3 acts as a negative regulator of cytokine or hormone signaling, but it is capable of positively regulating inflammatory responses in some cases, where it inhibits STAT3. Aberrant expression levels of SOCS3/STAT3 have been found in various bone marrow and lymphocytes, as well as in different nonhematopoietic cells, indicating their involvement in various infectious and inflammatory diseases.

CXCL8 is a well-studied proinflammatory chemokine that has been extensively researched. Neutrophil-activating factor (NAF) was discovered in the late 1980s by Peveri et al., and it was found to activate neutrophil exocytosis and oxidative burst through cell surface receptors in LPS-stimulated blood monocytes [22]. After its isolation and sequencing, NAF was named interleukin-8 (IL8) and CXCL8 due to the identification of other chemokines [23–25]. CXCL8 is released by various cells, such as monocytes [23, 25, 26], T lymphocytes [27, 28], macrophages, synovial cells [29], and keratinocytes [30], after appropriate stimulation. CXCR1 and CXCR2 are the two well-known receptors for CXCL8 that are expressed not only on leukocytes but also on other cell types, such as endothelial cells, smooth muscle cells, and fibroblasts. Evidence suggests that the activation of these receptors may contribute to several actions, including angiogenesis [31]. Numerous studies have indicated that CXCL8 and other IL-8 family chemokines induce endothelial cell chemotaxis in vitro and angiogenesis in vivo [32]. Therefore, CXCL8 might play a critical role in the early synovitis of arthritis that is characterized by vascular proliferation in the synovial membrane where CXCL8 can exert its effect.

This study aimed to further investigate the correlation between three effective biomarkers (JUN, SOCS3, CXCL8) and immune infiltrating cells, which play a crucial role in rheumatoid arthritis (RA), specifically in RF-negative pJIA and oJIA. The results showed a positive correlation between JUN and CXCL8 expression levels and the abundance of B-cell naive and T follicular helper cells. The term T follicular helper cells was coined in a series of studies on human tonsillar germinal center (GC) CD4 + T cells and CXCR5 + CD4 + T cells in the blood [33]. Previous research has demonstrated that T follicular helper cells are critical in RA progression and are linked to ectopic lymphoid structures (ELSs) in the joints, which consist of B cells, CD4 + T cells, and GCs [34]. CXCL13-expressing CD4 + T cells are associated with ELSs in joints [35], and GC-Tfh cells are the primary source of CXCL13 in human lymphoid tissue [36]. Therefore, Tfh cells are believed to contribute to the development of ELSs and support the autoantibody response of B cells. However, the biology of the interaction between Tfh cells and ELSs requires further exploration.

The present study has several limitations, as it relied solely on gene transcriptome analysis and lacked multiomics experiments. Furthermore, the data analysis was conducted using only the bioinformatics commit method, and subsequent in vivo and in vitro validation experiments are required to verify the findings. To overcome these limitations, future studies will incorporate a multiomics approach. Additionally, the next study aims to investigate the differences in arthritis between oJIA species with and without uveitis.

In summary, this study successfully identified six critical genes (JUN, SOCS3, CXCL8, KRAS, NFKBIA, and PTGS2) as biomarkers for the early diagnosis of RF-negative pJIA and oJIA. Furthermore, the study provided insights into the landscape of immune cells associated with RF-negative pJIA and oJIA and suggested that Tfh cells may contribute to the early onset of both RF-negative pJIA and oJIA.

## Conclusions

In the International League Against Rheumatism’s JIA classification, RF-negative pJIA and oJIA are the two JIA subtypes specific to children. In this study, we identified JUN, CXCL8, SOCS3, and KRAS as biomarkers for RF-negative pJIA and JUN, CXCL8, SOCS3, PTGS2, and NFKBIA as biomarkers for oJIA. These biomarkers can serve as valuable tools for disease diagnosis.Gene expression signatures associated with RF-negative pJIA and oJIA were discovered, thus helping to distinguish between these two distinct subtypes and contributing to accurate diagnosis and treatment selection.The new classification criteria for PRINTO juvenile idiopathic arthritis categorizes RF-negative pJIA and oJIA as the same subtype, and from our results, RF-negative pJIA and oJIA exhibit similarities in biomarkers and immune infiltration, validating this classification criteria.The findings on immune infiltration highlight the potential involvement of follicular helper T cells, CXCL8, JUN, and SOCS3 in the underlying mechanisms and biological processes of the disease. These findings contribute to a more comprehensive understanding of the disease development process and offer novel targets and strategies for disease treatment and drug development.

### Supplementary Information

**Additional file 1.**

## Data Availability

The datasets presented in this study can be found in online repositories. The names of the repository/repositories and accession number(s) can be found in the article.

## Abbreviations

JIA: Juvenile idiopathic arthritis
RF: Rheumatoid factor
pJIA: Polyarticular juvenile idiopathic arthritis
oJIA: Oligoarticular juvenile idiopathic arthritis
DEGs: Differentially expressed genes
WGCNA: Weighted gene co-expression network analysis
GEO: Gene Expression Omnibus
ILAR: The International League Against Rheumatism
GO: Gene Ontology
KEGG: The Kyoto Encyclopedia of Genes and Genomes
BP: Biological process
CC: Cellular component
MF: Molecular function
PPI: Protein‒protein interaction
RA: Rheumatoid arthritis
GC: Germinal center
ELSs: Ectopic lymphoid structures
